# Reassessing the phylogenetic relationships of *Pseudosorghum* and Saccharinae (Poaceae) using plastome and nuclear ribosomal sequences

**DOI:** 10.1016/j.pld.2025.03.002

**Published:** 2025-03-15

**Authors:** Kai Chen, Yan-Chun Liu, Yue Huang, Xu-Kun Wu, Hai-Ying Ma, Hua Peng, De-Zhu Li, Peng-Fei Ma

**Affiliations:** a State Key Laboratory of Plant Diversity and Specialty Crops, Kunming Institute of Botany, Chinese Academy of Sciences, Kunming, Yunnan 650201, China; b Germplasm Bank of Wild Species & Yunnan Key Laboratory of Crop Wild Relatives Omics, Kunming Institute of Botany, Chinese Academy of Sciences, Kunming, Yunnan 650201, China; c University of Chinese Academy of Sciences, Beijing 100049, China; d Shanghai Botanical Garden, Shanghai 200030, China; e Shanghai Engineering Research Center of Sustainable Plant Innovation, Shanghai 200231, China; f School of Ecology and Environmental Science, Yunnan University, Kunming, Yunnan 650504, China; g CAS Key Laboratory for Plant Diversity and Biogeography of East Asia, Kunming Institute of Botany, Chinese Academy of Sciences, Kunming, Yunnan 650201, China

**Keywords:** Phylogenomics, Saccharinae, *Pseudosorghum*, Plastome, nrDNA, Biogeography

## Abstract

The sugarcane subtribe Saccharinae (Andropogoneae, Poaceae) was established in 1846, but its delimitation has long been debated. Moreover, the relationships among the genera of Saccharinae remain unclear, and there is no consensus on whether *Pseudosorghum*, a small genus in tropical Asia with only two species, should be included. Here, we performed phylogenomic analyses using whole plastomes (69 of them newly sequenced) from 132 individuals, representing 65 species in 19 related genera. We also built trees with nuclear ribosomal DNA sequences. Our results justify the inclusion of *Pseudosorghum*, likely also the *Eulalia* Clade III, in Saccharinae. Furthermore, both morphological and molecular analyses support merging the two *Pseudosorghum* species. The backbone relationships of the Saccharinae phylogeny were highly supported with four polyphyletic clades of *Miscanthus* and the inclusion of *Narenga* and *Tripidium rufipilum* in *Saccharum*. *Pseudosorghum* is moderately supported as sister to the *Miscanthus* Clade I, while the remaining *Tripidium* species could be excluded from the subtribe. Saccharinae is estimated to have originated ∼3.73 million years ago in East Asia, followed by intercontinental dispersals. Our study provides a comprehensive phylogenetic framework for future taxonomic revisions of this economically important subtribe.

## Highlights

• A comprehensive phylogenetic framework was proposed for the important grass subtribe Saccharinae.
• Justify the phylogenetic position of *Pseudosorghum* in Saccharinae, as well as the merging of its two species.
• The inclusion of *Narenga* and *Tripidium rufipilum* in *Saccharum* was supported.
• Saccharinae may have originated in East Asia and experienced several dispersals and vicariance events during the Pleistocene.

## 1 Introduction

The subtribe Saccharinae, which includes many important sugar and biofuel crop species such as sugarcane (*Saccharum* spp.) and *Miscanthus* species (Brosse et al., 2012, Kellogg, 2015), was first proposed by Kunth (1815) as Saccharina. However, this name was invalid due to its misplaced rank as a section within the grass family (Poaceae). The validation of this name was attributed to Grisebach (1846). With a long history, delimitation of Saccharinae has varied greatly, being a hotspot of controversy in the grass taxonomy (Bentham, 1881; Hackel, 1889; Keng, 1939, 1959; Bor, 1970; Clayton, 1972; Clayton and Renvoize, 1986; Liu, 1997). Before the adoption of molecular evidence, the widely accepted classification of Saccharinae was provided by Clayton and Renvoize (1986) with 13 genera: *Spodiopogon* Trin., *Saccharum* L., *Eriochrysis* P. Beauv., *Miscanthus* Anderss., *Imperata* Cyr., *Eulalia* Kunth, *Homozeugos* Stapf, *Polytrias* Hack., *Lophopogon* Hack., *Pogonatherum* P. Beauv., *Eulaliopsis* Honda, *Microstegium* Nees, and *Polliniopsis* Hayata. However, such classification of Saccharinae was found to be polyphyletic and the genera included varied among different molecular phylogenetic studies (Hodkinson et al., 2002a; Lloyd Evans et al., 2019; Vorontsova et al., 2020; Welker et al., 2020a, 2020b). Moreover, several genera of Saccharinae were also found to be polyphyletic, such as *Eulalia* and *Tripidium* H. Scholz (syn. *Ripidium* Trin.). Once treated as a synonym under *Saccharum* or a section under *Erianthus* Michx., *Tripidium* is now recognized as a distinct genus (Welker et al., 2015, 2019; Lloyd Evans et al., 2019; Vorontsova et al., 2020), being excluded from Saccharinae with uncertain relationships in the tribe Andropogoneae (Soreng et al., 2022), possibly sister to the subtribe Rottboelliinae (Welker et al., 2020a).

A recent phylogenetic study based on plastid genomes and broad taxon sampling placed only two of the traditional 13 genera of Saccharinae, *Saccharum* and *Miscanthus*, within the subtribe, while including *Pseudosorghum* A. Camus (previously Sorghinae) (Welker et al., 2020a). This classification was adopted by Soreng et al. (2022) in an updated global phylogenetic classification of Poaceae. The remaining genera of Saccharinae have been classified into the other subtribes of Andropogoneae or treated as *incertae sedis* by Soreng et al. (2022). Therefore, the currently recognized Saccharinae contains about 46 species from three genera (POWO, 2024) in Andropogoneae, which comprises approximately 92 genera and more than 1200 species (Soreng et al., 2022). Among the 14 subtribes of Andropogoneae, Saccharinae is sister to Sorghinae, which includes *Sorghum* Moench and *Sarga* Ewart, among others (Soreng et al., 2022).

In Andropogoneae, spikelets are usually arranged in pairs, one sessile and bisexual, and one pedicellate and staminate or neuter in general (Skendzic et al., 2007; Kellogg, 2015). The similarity of shape and sex between sessile and pedicelled spikelets has long been regarded taxonomically important, and the Saccharinae species have traditionally been considered morphologically primitive in this tribe because their paired bisexual spikelets are alike (Bentham, 1881; Hackel, 1889; Keng, 1939, 1959; Bor, 1970; Clayton, 1972; Clayton and Renvoize, 1986; Liu, 1997). However, the inclusion of *Pseudosorghum*, which has dissimilar spikelet pairs, into Saccharinae casts doubt on this assumption (Clayton and Renvoize, 1986). *Pseudosorghum* is a small genus distributed in tropical Asia with two species (Camus, 1920), i.e., *P*. *fasciculare* (Roxb.) A. Camus and *P*. *zollingeri* (Steud.) A. Camus. *Pseudosorghum* is morphologically similar to *Sorghum* and *Bothriochloa* Kuntze (Clayton and Renvoize, 1986; Skendzic et al., 2007). The clustering of *Pseudosorghum* into Saccharinae would indicate that the characteristic of paired similar bisexual spikelets may no longer be a synapomorphy of this subtribe.

As important as the phylogenetic position of *Pseudosorghum* is*,* it has rarely been sampled. It was first included in a phylogenetic study in 2011 and found to be closely related to some species of *Eulalia*, *Miscanthus* and *Saccharum*, although the grouping was not clearly delimited (Teerawatananon et al., 2011). A more recent phylogenetic analysis, including 50 Andropogoneae species (Arthan et al., 2017), grouped *P*. *fasciculare* with *Eulalia siamensis* Bor. However, a subsequent study that used the same two accessions of *P*. *fasciculare* found that *Pseudosorghum* is sister to a clade of *Saccharum* and *Miscanthus* (Welker et al., 2020a). This discrepancy, based on the same two accessions of *P*. *fasciculare*, highlights the elusive relationship of this genus in Saccharinae. Clarifying it will also help to delimit the Saccharinae and to illuminate the evolutionary relationships within the subtribe.

*Pseudosorghum* has barely been examined in field investigations or morphological studies on herbarium specimens. For example, there are only two correctly identified specimens of *P*. *fasciculare* in the Chinese Virtual Herbarium (CVH, https://www.cvh.ac.cn/). This genus has not been collected in the field for almost four decades in China (the last voucher specimen 90627, YUKU, collected in 1990). When establishing the genus, Camus (1920) suggested *P*. *fasciculare* was distinct from *P*. *zollingeri* based on the number of spikelet pairs per raceme (3–6 spikelet pairs in *P*. *fasciculare* vs. 10–14 spikelet pairs in *P*. *zollingeri*) and the state of pedicellate spikelets (barren pedicelled spikelets without any lemmas or paleas *vs*. male pedicelled spikelets with lemmas). In the grass accounts of *Flora of China* (*FOC*), Chen et al. (2006) overturned the classification of the two species in *Pseudosorghum* with *P*. *zollingeri* reduced to a synonym of *P*. *fasciculare* but without any taxonomic justification. Thus, it remains unclear whether there are one or two species in *Pseudosorghum*.

Over the past decades, phylogenomics has become a conventional approach for phylogenetic reconstruction and classification of plants with the development of sequencing technologies (Delsuc et al., 2005; Fu et al., 2022; Guo et al., 2023). Among them, plastid phylogenomics has broadly been used in successfully resolving the phylogenetic relationships at different taxonomic levels, from the family, tribe, genus, and even to the species (Ma et al., 2014; Huang et al., 2022; Wu et al., 2022; Zhou et al., 2022; Lv et al., 2023; Chen et al., 2024; Gu et al., 2024). By employing the method of genome skimming, we can efficiently extract highly repetitive genomic regions from plant cells, including plastid genome and nuclear ribosomal DNA (nrDNA) sequences (Straub et al., 2012; Zhang et al., 2023). Here, by utilizing this kind of molecular evidence, we aimed to: 1) examine the phylogenetic placement of *Pseudosorghum* with more comprehensive taxon sampling from closely related genera; 2) re-evaluate the delimitation of the Saccharinae subtribe; and 3) enhance our understanding of the evolutionary history of the Saccharinae species by estimating their divergence times and biogeographic patterns.

## 2 Materials and methods

### 2.1 *Taxon sampling and morphological analysis of* Pseudosorghum

To accurately estimate phylogenetic relationships within Saccharinae, we sampled a total of 159 individuals representing 19 genera and 65 species of the subtribe and its close relatives, derived from updated phylogenies of Andropogoneae (Soreng et al., 2022). Specifically, we focused on genera that are morphologically or phylogenetically related to *Pseudosorghum*, including *Bothriochloa* (5 taxa/10 accessions), *Eulalia* (6/9), *Miscanthus* (13/35), *Narenga* (1/5), *Saccharum* (4/15), *Sorghum* (4/11), and *Tripidium* (5/17), as well as other genera, including *Andropogon* (7/14), *Apluda* (1/1), *Capillipedium* (4/5), *Dichanthium* (3/5), *Hemisorghum* (1/1), *Heteropogon* (3/7), *Lasiorhachis* (1/2), *Rottboellia* (1/3), *Sarga* (1/2), and *Themeda* (2/5). Following Welker et al. (2020a), *Coix lacryma-jobi* L. was selected as the outgroup. A total of 69 individuals were newly sampled, while sequence data from the remaining 90 were downloaded from NCBI (https://www.ncbi.nlm.nih.gov/). Detailed information on the sampled taxa, voucher specimens, and sources of data are provided in Table S1.

Delimitation of *Pseudosorghum* species was based on specimens collected in the field and those in herbaria. Twelve *P*. *zollingeri* specimens were found at the YUKU herbarium and nine *P*. *fasciculare* specimens were newly collected in Zhenkang County, Yunnan Province, China (Table S2). Diagnostic features used to distinguish between the two *Pseudosorghum* species included the number of spikelets in the inflorescence and the absence/presence of hyaline lemmas in the pedicelled spikelets (Camus, 1920; Clayton and Renvoize, 1986; Sun, 2003; Chen et al., 2006). These diagnostic features were examined across a total of ten samples of the *P*. *fasciculare* and *P*. *zollingeri* specimens (Table S2).

### 2.2 Sequencing, assembly and gene annotation

Total genomic DNA from newly sampled individuals was extracted from silica-dried leaves collected in the field or, in a few cases, from herbarium specimens, using a modified CTAB method (Doyle and Doyle, 1987). Purified DNA samples were sheared into 350 bp fragments to construct short-insert libraries for sequencing with 2 × 150 bp reads on a DNBSEQ-T7 platform. About 10 Gb of high-quality sequencing data was selected for each sample and assembled into the plastid genome and nrDNA sequences using the GetOrganelle pipeline (Jin et al., 2020).

The resulting plastomes were annotated using the Plastid Genome Annotator (PGA) (Qu et al., 2019) and manually checked and corrected, if necessary, in Geneious v.9.0.2 (Kearse et al., 2012). Due to the potential errors in gene annotations, the downloaded plastomes were also reannotated with the same procedure. A plastome map of *Pseudosorghum* was drawn with OrganellarGenomeDRAW (OGDRAW) v.1.3.1 (Greiner et al., 2019). The nrDNA sequence of *Oryza sativa* L. (GenBank Accession Number: KM036285) was used as the reference to assemble and annotate nrDNA sequences for the newly sequenced samples.

### 2.3 Phylogenetic analyses, split and haplotype networks

For phylogenetic analyses, we also used Geneious to extract the coding sequences of 78 protein-coding genes (CDS), large single copy (LSC), small single copy (SSC), and inverted repeat (IR) regions. In addition, ITS regions were subsampled from nrDNA sequences. These assembled datasets were individually aligned by MAFFT v.7.22 using the default setting (Katoh and Standley, 2013). The resulting alignments were trimmed using trimAl with default parameters to remove ambiguously aligned regions (Sanchez et al., 2011).

A maximum likelihood (ML; Felsenstein, 1973) tree was constructed using raxmlGUI v.2.0.10 (Edler et al., 2021). One thousand rapid bootstrap replicates were conducted with GTRGAMMAI as the default model. For Bayesian inference (BI), we selected the best-fitting model for each dataset using Bayesian Information Criterion (BIC) by ModelFinder (Kalyaanamoorthy et al., 2017). Detail information on the data matrix and model is provided in Table 1. Bayesian analysis was performed for 60 million generations using MrBayes v.3.2.7 (Ronquist and Huelsenbeck, 2003), with the first 25% of the trees discarded as burn-in. All the runs reached convergence with an average standard deviation of the split frequency smaller than 0.01. The remaining trees were used to calculate a 50% majority-rule consensus tree with Bayesian posterior probabilities (BPP). Visualization of phylogenetic trees was conducted using FigTree v.1.4.4 (Rambaut, 2018) and tvBOT (Xie et al., 2023).

**Table 1:** Characteristics of different datasets used in this study.

Datasets		Number of samples (newly generated)	Number of taxa	Alignment length (bp)	Variable sites (bp) (%)	Parsimony informative sites (bp) (%)
Plastome	132 (69)		65	140,756	9079 (6.45%)	6208 (4.41%)
CDS	132 (69)		65	56,554	2729 (4.83%)	1927 (3.41%)
LSC	132 (69)		65	82,667	7085 (8.57%)	4960 (6.00%)
SSC	132 (69)		65	12,532	1087 (8.67%)	795 (6.34%)
IR	132 (69)		65	22,776	467 (2.05%)	239 (1.05%)
nrDNA	63 (62)		30	5783	515 (8.91%)	372 (6.43%)
ITS	86 (59)		38	584	211 (36.13%)	180 (30.82%)

To identify potential conflicting phylogenetic signals within the plastid genome sequences, split-decomposition network analysis was conducted using SplitsTree CE (Huson and Bryant, 2006) with “hamming distances ambiguous states” and “neighbor net”. We further conducted a haplotype network analysis of the closely related species of *Pseudosorghum*, mostly sampled with three or more individuals, from the *Miscanthus*, *Narenga*, and *Saccharum*, as well as the *Eulalia* Clade III and *Tripidium rufipilum*. Haplotypes were extracted using DnaSP v.6 (Rozas et al., 2017), and the haplotype network was constructed using PopART v.1.7 with the TCS method (Leigh and Bryant, 2015).

### 2.4 Molecular dating and biogeographic analysis

To obtain a time-calibrated evolutionary framework for Saccharinae, divergence times were estimated based on the plastid genome sequences by treePL (Smith and O'Meara, 2012; Maurin, 2020). The optimal smoothing value of the final treePL analysis was determined by the lowest χ^2^ value through cross-validation tests with 1e+32 selected (Table S3). Due to the lack of a fossil record, we employed four secondary calibration points from TimeTree and previous studies on Andropogoneae (Kumar et al., 2017, 2022; Hackel et al., 2018; Gallaher et al., 2019). The estimated divergence time between the ancestor of *Coix lacryma-jobi* and the ancestor of *Rottboellia cochinchinensis* (Lour.) Clayton is set to 5.4–9.2 million years ago [Ma] (95% Confidence Interval; CI) (indicated as T1). Based on the synonymous substitution rates (Ks) inferred from nuclear DNA sequences (Kim et al., 2014; Zhang et al., 2018, 2021), another three divergence time points were set in the treePL analysis, i.e., divergence between the ancestor of *Sorghum* and the ancestor of *Miscanthus* (3.8–4.6 Ma, T2), *Miscanthus* and *Saccharum* (3.1–4.1 Ma, T3), and *Saccharum officinarum* and *Sac*. *spontaneum* (0.85–1.05 Ma, T4).

For biogeographic analysis, the tree derived from the treePL analysis above was used. Species distribution data was obtained from the Global Biodiversity Information Facility (GBIF, https://www.gbif.org/), Plants of the World Online (POWO, 2024), and *FOC* (Chen et al., 2006). We delimited eight biogeographic regions for analysis following Welker et al. (2020a): (A) Africa; (B) Central America; (C) East Asia; (D) Europe; (E) North America; (F) Oceania; (G) South America; (H) West Asia. Historical distributions were reconstructed using RASP v.4.2 (Ree and Smith, 2008; Yu et al., 2015). Six biogeographic models provided by BioGeoBEARS (Matzke, 2018), i.e., DEC, DEC + J, DIVALIKE, DIVALIKE + J, BAYAREALIKE, and BAYAREALIKE + J, were tested to select the best-fit using log-likelihood (LnL) under the AICc and AICc_wt criteria (Massana et al., 2015). The number of maximum areas for ancestral nodes was set to eight, covering all distribution regions for the sampled species.

## 3 Results

### 3.1 *Morphological analysis and plastomes of* Pseudosorghum

**Fig. 1:**
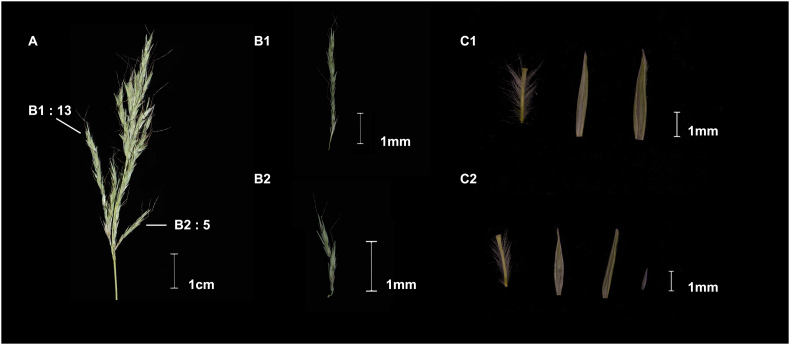
Inflorescence and floral traits of one individual specimen of *Pseudosorghum*. A, Inflorescence composed of racemes; B1, An intact raceme (from A) with 13 nodes/spikelet pairs; B2, An intact raceme (from A) with five nodes/spikelet pairs; C1, A pedicelled spikelet (from B1), showing two subequal glumes without lemma; C2, A pedicelled spikelet (from B2), showing a tiny lemma enclosed by two subequal glumes.

After thorough field investigations across all the recorded localities of *Pseudosorghum* specimens in China (Fig. S1), we only managed to collect living *Pseudosorghum* plants from a small population in Zhenkang County, Yunnan Province. These plants were found on the edges of agricultural fields under intense human disturbance. Racemes of individual plants had either 5–6 nodes or 6–14 nodes (Fig. 1A–B), a diagnostic character previously used to distinguish the two putative *Pseudosorghum* species (Camus, 1920; Clayton and Renvoize, 1986; Sun, 2003). A detailed examination of more specimens (Table S2) indicated that the numbers of nodes overlapped completely in *P. fasciculare* and *P. zollingeri*. We also observed that the pedicelled spikelets from the same specimen could have the hyaline lemma or not (Fig. 1C). These results support the acceptance of a single species, consistent with the taxonomic treatment of *Pseudosorghum* in *FOC* (Chen et al., 2006).

We assembled a total of 69 new plastid genomes, 65 of which were complete. All plastomes had the typical quadripartite structure. Genome size and structure of the five newly assembled plastomes of *Pseudosorghum*, as well as to those of two published ones (NC_035024 and KY596157), are remarkably similar (Fig. S2). The total length of these seven plastomes ranged from 140,347 bp to 140,561 bp, encoding an identical set of 78 unique protein-coding genes, 30 tRNA genes, and four rRNA genes.

### 3.2 Characteristics of molecular datasets

We used 132 samples from 65 species to assemble datasets of plastome, CDS, as well as LSC, SSC and IR regions (Table 1). After alignment and trimming, the plastome was 140,756 bp; CDS, 56,554 bp; LSC, 82,667 bp; SSC, 12,532 bp; and IR, 22,776 bp. The plastome dataset had the highest number of parsimony-informative (PI) sites (6208, 4.41%), whereas IR region had the lowest proportion of PI sites (239, 1.05%), as expected.

We successfully retrieved 62 nrDNA sequences (5594–5789 bp) as well as 59 ITS sequences (579–593 bp) from the sequencing data of 66 samples. The final alignment of 63 nrDNA sequences, for which there is much less available data from Saccharinae and related taxa, was 5783 bp, with 372 PI sites. The ITS dataset comprised 86 sequences with an alignment of 584 bp and 180 PI sites.

### 3.3 Phylogenetic reconstruction

**Fig. 2:**
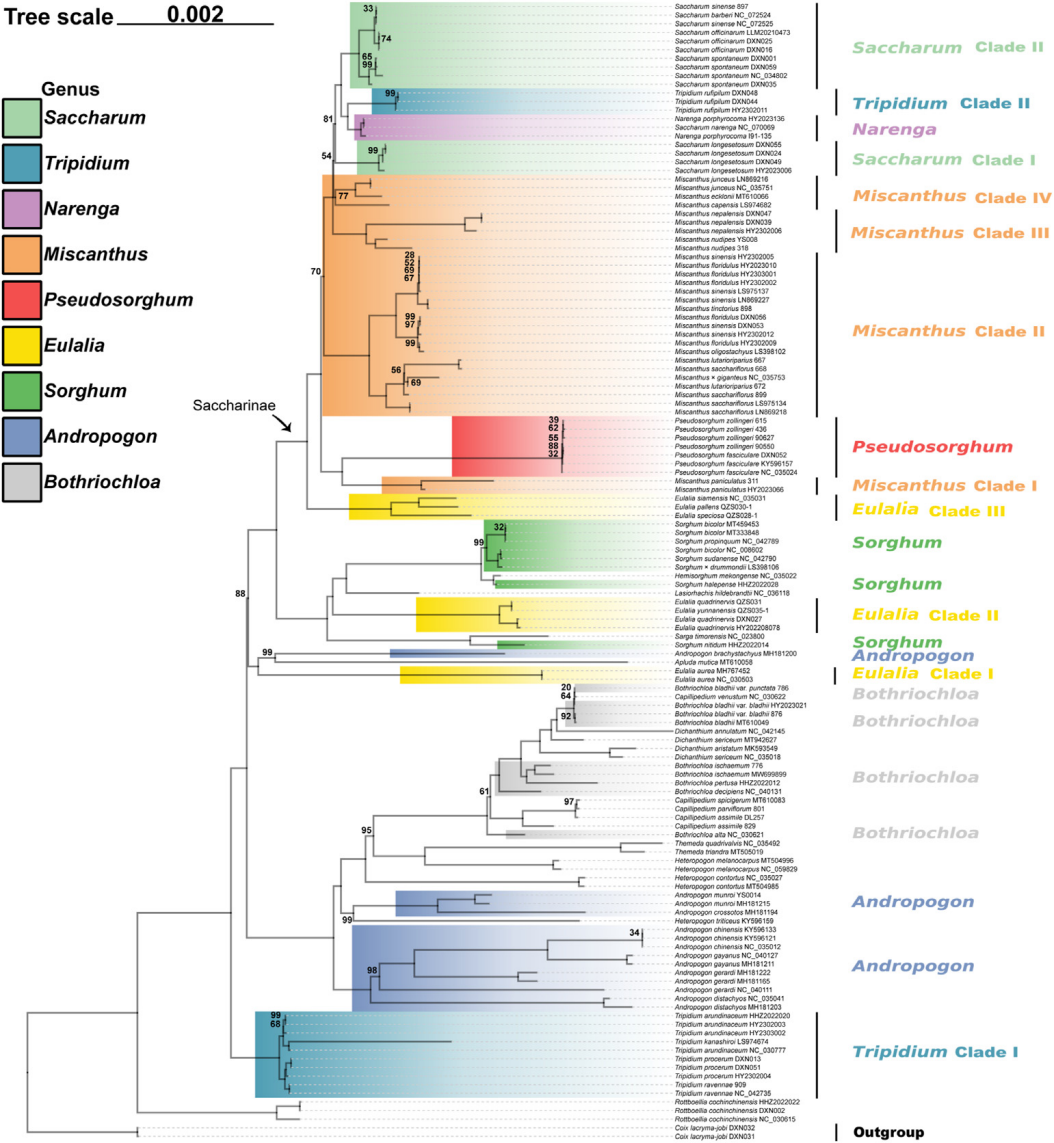
Maximum likelihood (ML) phylogenetic tree based on whole plastome. Only MLBS values < 100% are shown. The corresponding genera/clades are labeled different colors.

**Fig. 3:**
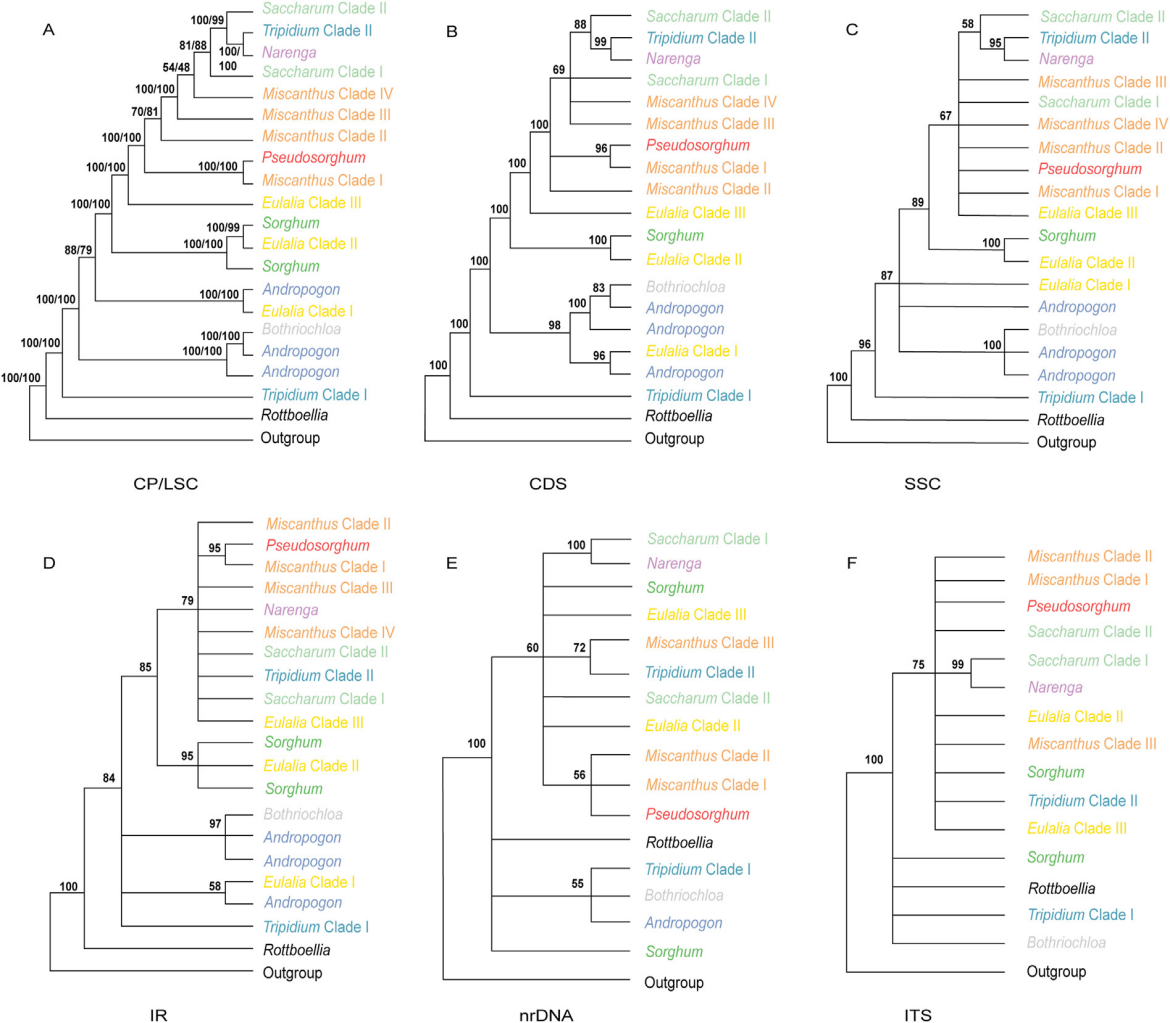
Summary of phylogenetic relationships among major lineages as recovered from plastomes, nrDNA and ITS regions using ML analyses. Phylogenetic relationships with MLBS values < 50% are collapsed. The corresponding genera/clades are labeled different colors following Fig. 2. A, Plastome and LSC region; B, CDS region; C, SSC region; D, IR region; E, nrDNA sequences; F, ITS sequences.

Bayesian and ML analyses of the whole plastome dataset generated the phylogenetic reconstruction with the highest resolution (Figs. 2 and S3–S7). Similar topologies were generated for the whole plastome and the other four datasets (Fig. 3A–D), although there were some differences in poorly supported nodes. Moreover, the two methods produced highly convergent phylogenetic relationships. Here, we focus on the best-resolved tree based on the whole genome sequences.

In most cases, the two or more accessions sampled for the same species formed monophyletic groups (Fig. 2). All of the seven samples of *Pseudosorghum* formed a 100% supported clade. However, the three samples previously identified as *P. fasciculare* and four identified as *P. zollingeri* did not form monophyletic clades exclusively. In fact, the phylogenetic resolution within *Pseudosorghum* was low with ML bootstrap support (BS) values mostly below 70%. Moreover, the seven samples of *Pseudosorghum* exhibited six distinct haplotypes, with a maximum step length of five (Fig. S8), and *P*. *zollingeri* 90550 and *P*. *fasciculare* DXN052 clustered together (Haplotype 6). The genetic differentiation among these haplotypes was found to be much lower compared to those within other closely related genera. Together, these results consistently support the recognition of a single species in *Pseudosorghum*.

Nearly all sampled allies of *Pseudosorghum* (i.e., *Andropogon*, *Bothriochloa*, *Eulalia*, *Miscanthus*, *Saccharum*, *Sorghum*, and *Tripidium*) were revealed as polyphyletic (Fig. 2). For ease of description, we divided and labeled some phylogenetic groups as different clades. Apart from three *Tripidium rufipilum* samples that were deeply embedded within *Saccharum*, the remaining four species of this genus, defined as the *Tripidium* Clade I diverged early in the tree. There was a large group consisting of samples mainly from the genera *Andropogon* and *Bothriochloa*, which was sister to the group of the remaining samples (MLBS = 100%; BPP = 1.0). The six species of *Eulalia* formed three highly supported clades. Among them, *Eulalia* Clade I (represented by *E. aurea*, the type species), together with *Apluda mutica* and *Andropogon brachystachyus*, diverged first. *Eulalia* Clade II (*E. quadrinervis* and *E. yunnanensis*) was clustered into *Sorghum* (MLBS = 100%; BPP = 1.0). *Eulalia* Clade III (*E. speciosa*, *E. siamensis*, and *E. pallens*) was sister to Saccharinae (MLBS = 100%; BPP = 1.0), and this relationship was fully supported in all plastid datasets except for the SSC and IR datasets with low to moderate support values (Fig. 3A–D).

In Saccharinae, *Pseudosorghum* was strongly supported as sister to *Miscanthus* Clade I (represented by *M. paniculatus* from *M.* subg. *Rubimons*; Chen et al., 2006; Liu and Peng, 2010) by different plastid datasets (Fig. 3A–D). The *Pseudosorghum* + *Miscanthus* Clade I was sister to the remaining sampled species within the subtribe, in which another three well-resolved clades of *Miscanthus* firstly diverged along the tree sequentially with moderate to strong support. The *Miscanthus* Clade II comprised *M.* sect. *Miscanthus* (Honda, 1930; Adati, 1962; Renvoize, 2003) and *M.* sect. *Triarrhena* (Honda, 1930; Renvoize, 2003), whereas Clade III and IV consisted of *M.* sect. *Diandra* (Keng, 1959; Lee, 1964a, 1964b, 1964c, 1964d; Chen et al., 2006) and *M.* sect. *Miscanthidium* (Pilger, 1940; Clayton, 1970), respectively. *Saccharum* species and their close relatives were grouped into four major clades: the *Saccharum* Clade I (*Sac. longesetosum*), *Narenga*, the *Tripidium* Clade II, and the *Saccharum* Clade II (including *Sac. sinense*, *Sac. officinarum*, and *Sac. spontaneum*). However, only the sister relationship between *Narenga* and the *Tripidium* Clade II was highly supported in most cases (Fig. 3A–D).

Our construction of a phylogenetic network largely reflected the relationships described above (Fig. S9). Specifically, the identified major clades were also well resolved. Nevertheless, clear conflicting phylogenetic signals were observed around the moderately supported nodes, especially those connecting the *Tripidium* Clade I and within *Saccharum*.

**Fig. 4:**
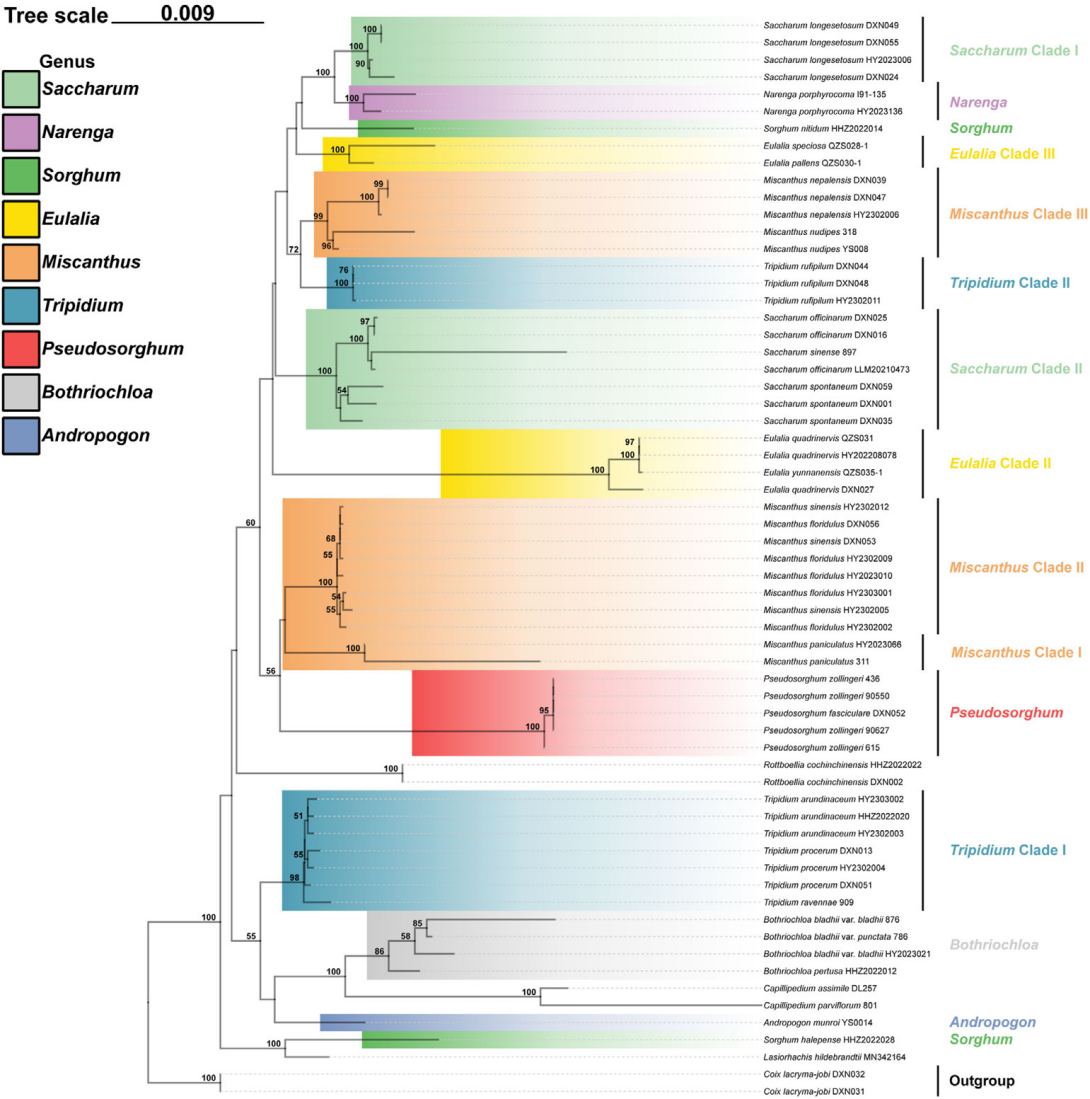
ML phylogenetic tree based on nrDNA sequences. Only MLBS values ≥ 50 % are shown. The corresponding genera/clades are labeled different colors following Fig. 2.

We built phylogenetic trees based on the nrDNA and ITS datasets (Fig. 3E and F), although with less taxon sampling for nrDNA sequence data (Table 1). Phylogenetic trees based on nuclear data were poorly resolved, with low support values (MLBS < 50% and BPP < 0.7) (Figs. 4, S10 and S11), particularly along the backbone nodes. Nevertheless, the support values for the shallow relationships were moderate, with the clades in the plastid phylogenies largely identified. For example, the samples of *Miscanthus* and *Tripidium* were similarly clustered into three and two independent clades, respectively. The monophyly of *Pseudosorghum* was strongly supported (MLBS = 100%; BPP = 1.0), while the samples previously identified as *P. fasciculare* and *P. zollingeri* did not form separate clades either. However, the sister relationship between *Pseudosorghum* and the *Miscanthus* Clade I was not resolved. The grouping of *Pseudosorghum* + *Miscanthus* + *Saccharum* + *Narenga* + the *Tripidium* Clade II corresponding to Saccharinae was still recovered despite moderate support, and with some *Eulalia* and *Sorghum* species within the clade.

### 3.4 Divergence time estimation

**Fig. 5:**
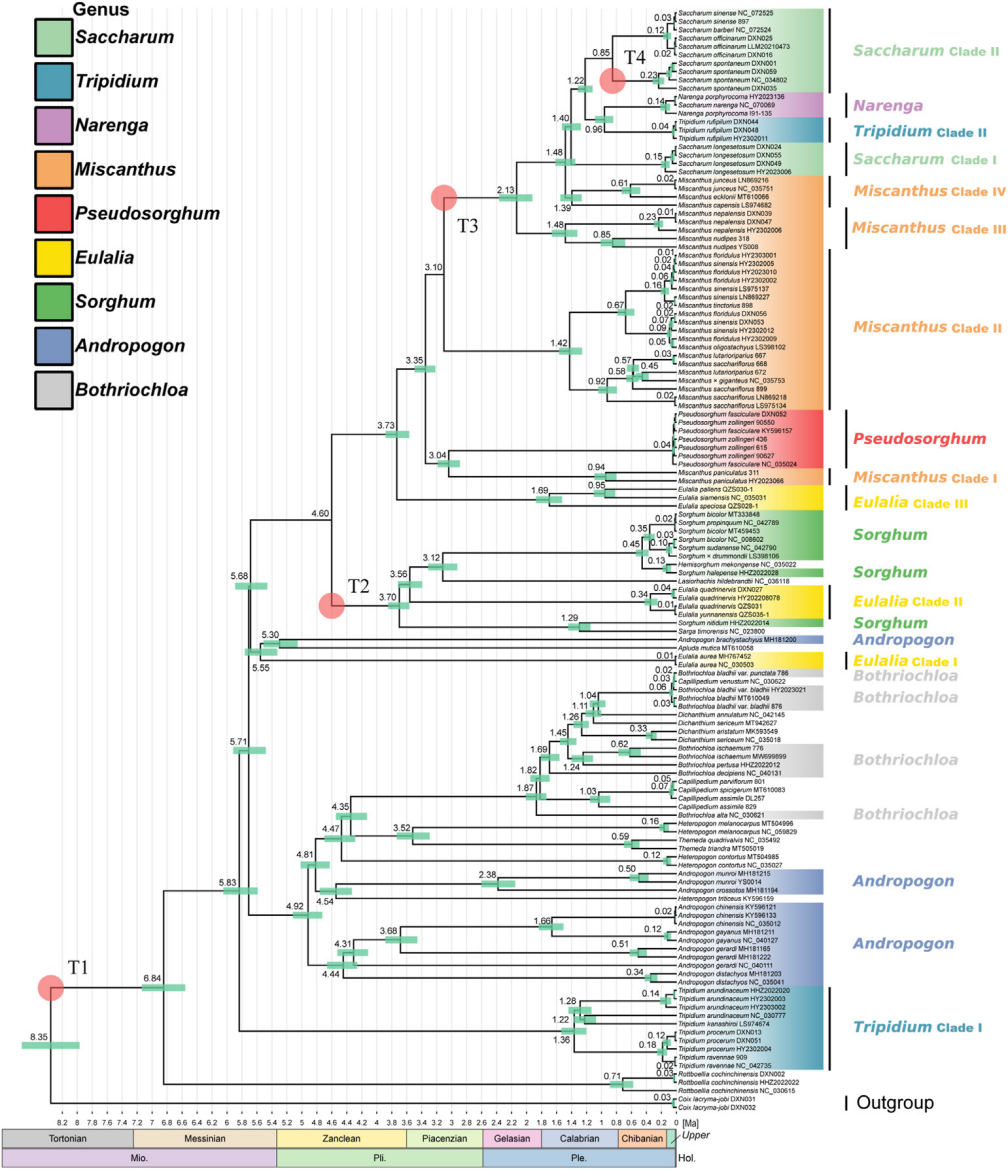
The time-calibrated tree of Saccharinae and its relatives. Red circles indicate four secondary calibration time points. Green node bars show the estimates of the median age in million years ago (Ma) and 95% CI.

Our results indicate that Saccharinae had an origin in the Zanclean period of Pliocene around ∼3.73 million years ago (Ma) (95% CI = ∼3.56–3.88 Ma) (Fig. 5). The subtribe began to diversify with the emergence of *Pseudosorghum* at ∼3.04 Ma (95% CI = ∼2.89–3.18 Ma). However, the diversification of this genus occurred very recently (∼0.04 Ma; 95% CI = ∼0.03–0.06 Ma), reflecting the limited genetic difference among the sampled individuals. For the other clades of Saccharinae, the diversification occurred much earlier, concentrating around 1.40 Ma. Moreover, species divergence was concentrated from the Upper Pleistocene to Chibanian Age.

For the groups supposed to be closely related to *Pseudosorghum*, the estimated crown ages were as followed: ∼1.36 Ma for the *Tripidium* Clade I (95% CI = ∼1.20–1.53 Ma), ∼1.87 Ma for the aggregated assemblage of *Bothriochloa* (95% CI = ∼1.73–2.01 Ma), ∼3.70 Ma for the group of *Sorghum* (95% CI = ∼3.56–3.85 Ma), and ∼1.69 Ma for the *Eulalia* Clade III (95% CI = ∼1.52–1.87 Ma). The majority of these groups diverged from *Pseudosorghum* more than 4.60 Ma.

### 3.5 Biogeography

**Fig. 6:**
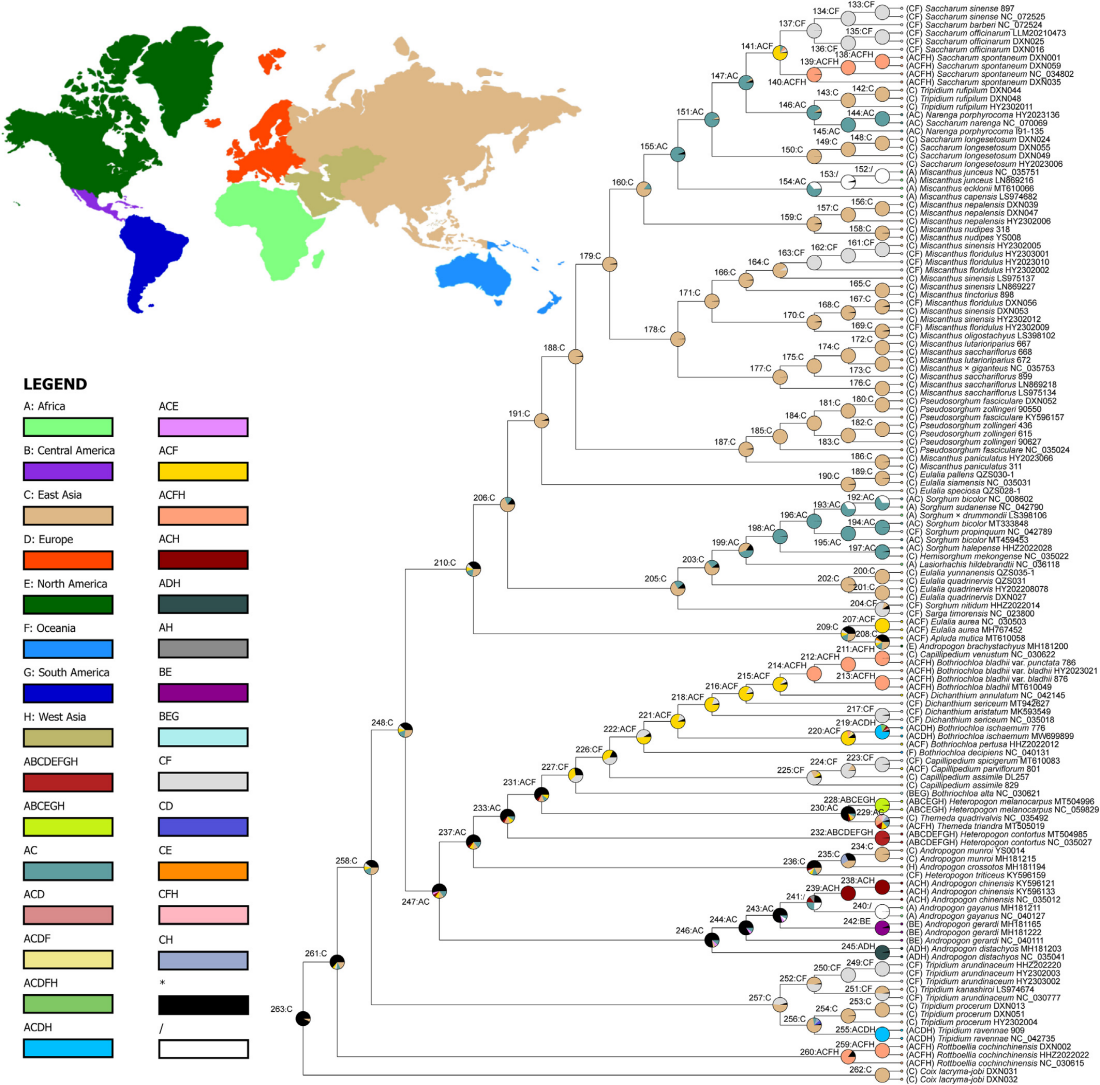
Ancestral area reconstruction for Saccharinae and its relatives using RASP, combined with species distribution information from *FOC*, GBIF and POWO. The pie chart represents the frequency of ancestral areas. Multi-areas are colored in black while any ancestral region that occurs in at least one node with a frequency below 5% is white.

BAYAREALIKE was selected as the best model for analysis. The general topological structure was similar to the best tree inferred from the whole plastome. Our analysis indicated that the common ancestor of Saccharinae and the *Eulalia* Clade III originated in East Asia (94.62% at node 191) (Fig. 6), as well as did the ancestors of *Pseudosorghum* and the rest of Saccharinae (97.83% and 97.87% at nodes 187 and 188, respectively).

Saccharinae experienced several major species dispersal events: the common ancestor of the *Miscanthus* Clade IV, *Narenga* and *Saccharum* from area C (East Asia; 85.72% at node 160) into areas AC (Africa + East Asia; 94.09% at node 155), the ancestor of the *Saccharum* Clade II from areas AC (Africa + East Asia; 89.17% at node 147) into ACF (Africa + East Asia + Oceania; 75.37% at node 141), and *Sac. spontaneum* from areas ACF into ACFH (Africa + East Asia + Oceania + West Asia; 99% at node 140). These events all occurred during the Pleistocene. In addition, the lineage of *M. floridulus* was dispersed from area C (East Asia) into areas CF (East Asia + Oceania) recently. The distribution of the ancestor of *Sac. longesetosum* was restricted to C (East Asia; 99.80% at node 150) from the ancestral area of AC (Africa + East Asia; 93.49% at node 151), as was the ancestor of *Tripidium rufipilum*. Some species of the *Saccharum* Clade II were also dispersed in Africa, with the current distribution restricted to East Asia and Oceania (99.66% at node 137).

## 4 Discussion

With the advance of sequencing technology, phylogenomics has become an effective approach to address evolutionary questions (Bock et al., 2014; Zeng et al., 2018; Guo et al., 2021; Zhou et al., 2022; Lv et al., 2023; Hu et al., 2024). Here, we newly obtained the plastomes of 69 samples from the Saccharinae subtribe and its relatives by sequencing of total genomic DNA, totaling up to 132 samples of 65 species for the most comprehensive phylogeny of the subtribe to date. Moreover, several endemic species from China, such as *Miscanthus paniculatus* and *Eulalia yunnanensis*, were previously not included in the phylogenetic analyses of Saccharinae. We also sampled more than one individual for the majority of species for robust phylogenetic relationships. Increased taxon sampling and expanded DNA datasets reaffirm the delimitation of Saccharinae with strong support, providing a robust phylogenetic framework for its clades and genera. This work facilitates a deep understanding of the evolution of this economically important subtribe. Below, we discuss the implications of our results on the understanding of the taxonomy, systematics, and evolutionary history of Saccharinae.

The phylogenetic analysis presented here confirms that the genera *Pseudosorghum*, *Miscanthus* and *Saccharum* (including *Tripidium rufipilum* and *Narenga*) belong to this subtribe, largely consistent with the classification of Soreng et al. (2022). The difference is that our results indicate that *Narenga* is closely related to *Saccharum*, rather than considering *Narenga* as a synonym of *Miscanthus*. The three genera, along with the *Eulalia* Clade III, formed a strongly supported monophyletic clade sister to the subtribe Sorghinae. The *Eulalia* Clade III, with three sampled species, is more closely related to Saccharinae than to Sorghinae. However, the six sampled *Eulalia* species here (out of a total 34 recognized species) were grouped into three clades, while the other two clades fell into the Sorghinae or Apludinae subtribes, respectively. This is consistent with the consideration of *Eulalia s.l.* as apparently polyphyletic and *Eulalia s.s*. belonging to Apludinae (Soreng et al., 2022). Even though our results indicate that the *Eulalia* Clade III should be included in Saccharinae, future in-depth study that includes samples across the paleotropics and nuclear genomic data should be conducted to determine whether some of the *Eulalia* species can be transferred into Sorghinae. In addition, the three individuals of *Tripidium rufipilum* collected in China clustered into *Saccharum*, indicating that they belong to Saccharinae, paraphyletic to the other species of the genus. This differs from the results of Lloyd Evans et al. (2019) (probably caused by misidentification or mislabeling of the accession LS974679.1 from USDA with uncertain origin) but consistent with recent findings based on the whole nuclear genomes (Kui et al., 2023; Wang et al., 2023), where *Tripidium rufipilum* was suggested as the most closely related diploid relative of cultivated sugarcane.

Consistent with previous studies, our analysis did not support the monophyly of *Saccharum* or *Miscanthus*, the two major genera of Saccharinae (Hodkinson et al., 2002a, 2002b; Welker et al., 2020a, 2020b). *Saccharum* was resolved into two clades, and contained species of *Narenga* and one species of *Tripidium*. This finding suggests that *Saccharum* should be expanded to include *Narenga* as a synonym and its two species should be transferred into *Saccharum* (Chen et al., 2006). *Miscanthus* was resolved into four major clades, some of which have been previously suggested (Al-Janabi et al., 1994; Sobral et al., 1994; Hodkinson et al., 2002b; Vorontsova al., 2020; Welker et al., 2020a, 2020b; Vasquez et al., 2022). The circumscription of these clades largely corresponds to the division of subgenus or sections of *Miscanthus* based on morphology. For example, *Miscanthus* Clade II comprised *M.* sect. *Miscanthus* (Honda, 1930; Adati, 1962; Renvoize, 2003) and *M.* sect. *Triarrhena* (Honda, 1930; Renvoize, 2003), while Clade III aligns to *M.* sect. *Diandra* (Keng, 1959; Lee, 1964a, 1964b, 1964c, 1964d; Chen et al., 2006) and Clade IV to *M.* sect. *Miscanthidium* (Pilger, 1940; Clayton, 1970), although they did not form a monophyletic group. Instead, the four clades diverged successively in relation to *Saccharum*, calling for future in-depth study of *Miscanthus*.

In contrast to *Saccharum* and *Miscanthus*, *Pseudosorghum* remains understudied and is much less known, as well as species-poor, with two putative species documented (Camus, 1920; Sun, 2003). Moreover, evidence from morphological, haplotype and phylogenetic analyses all indicate that the two *Pseudosorghum* species should be merged into one, as suggested previously (Chen et al., 2006). Because the basionym of *P*. *fasciculare, Andropogon fascicularis* Roxb. had priority (Roxburgh, 1820) over *Andropogon zollingeri* Steud. (Steudel, 1854), the basionym of *P*. *zollingeri*, we confirm the taxonomic treatment of reducing *P*. *zollingeri* to a synonym of *P*. *fasciculare* in *FOC*.

Previously, *Pseudosorghum* was suggested to be morphologically more similar to *Sorghum* and *Bothriochloa* (Haines, 1924; Clayton and Renvoize, 1986; Skendzic et al., 2007; Fang, 1986). With multiple representative species of these genera included in the phylogenetic analyses, we can confidently conclude that *Pseudosorghum* is a member of Saccharinae as in previous study (Welker et al., 2020a). However, unlike being an early-diverging lineage of Saccharinae, *Pseudosorghum* was found to be sister to *Miscanthus* Clade I, represented by an endemic species of *Miscanthus paniculatus* from Southwest China, without clear morphological synapomorphy (details below). Additional data for these species, especially those from nuclear DNA sequences, are critically needed to further evaluate this enigmatic relationship. In addition, *P*. *fasciculare* may be diploid with 2*n* = 20 chromosomes (Harlan et al., 1962; Estep et al., 2014), potentially holding key information for disentangling the complex evolutionary history of polyploidy in Saccharinae. Taken together, *Pseudosorghum* may represent a missing piece of the evolutionary puzzle of Saccharinae, despite with a knowledge gap remained in the nuclear genome of this genus. This highlights the need for substantial conservation efforts for this genus, as our comprehensive filed survey found only two wild populations, which are narrowly distributed along the farmland of Zhenkang County in Yunnan Province and the habitat are prone to loss due to farming activities. The other historical locations surveyed are either covered by banana cultivation or being opened for farming with no living plants of *Pseudosorghum* found.

Conflicts between traditional morphology-based taxonomic treatments and phylogenies based on molecular evidence are widespread and also important to our understanding of plant evolution (Cox et al., 2014; Wang et al., 2017; Coiro et al., 2019). Specifically, the relationships within Saccharinae have not been reflected by morphological characteristics of spikelet pairs (Hodkinson et al., 2002a; Kellogg, 2015; Vorontsova et al., 2020; Welker et al., 2020a). However, the similarity of shape and sex between sessile and pedicelled spikelets has long been regarded as taxonomically important in the tribe Andropogoneae (Bentham, 1881; Hackel, 1889; Arthan et al., 2017; Welker et al., 2019, 2020b; Vasquez et al., 2022). *Pseudosorghum* had been arranged in Sorghinae because of dissimilar spikelet pairs, as other members of it; additionally, Saccharinae has been considered relatively primitive, with similar bisexual spikelet pairs (Hackel, 1889; Keng, 1939, 1959; Bor, 1970; Clayton, 1972; Clayton and Renvoize, 1986; Liu 1997). *Pseudosorghum* is morphologically distinct from *M*. *paniculatus* in many ways, such as the pattern and shape of inflorescences, the sex and structures of the spikelet pairs, the hairiness and texture of the glumes, and the structure of the upper lemma and awns. This would pose challenges for taxonomists to re-evaluate the importance of spikelet pairs in grass classification as Kellogg (2015) pointed out that the reduction of pedicellate spikelet occurred more than once and thus may not indicate phylogenetic relationships.

One possible explanation for the disparity between the morphology and molecular evolution in Saccharinae is the rapid diversification of lineages and species. The whole subtribe was estimated to have originated around ∼3.73 Ma in the Pliocene with the major clades diverging in a short time between ∼3.10 Ma and ∼1.40 Ma. These divergence time estimates are generally consistent with previous studies (Welker et al., 2020a), or slightly younger for certain nodes. The extensive speciation events have mainly occurred during and after the Pleistocene (Calabrian) period primarily in East Asia (extending to western New Guinea), with subsequent dispersals to Oceania (particularly eastern New Guinea) and expansion to West Asia and Africa. The spikelets of many taxa in Saccharinae have awns, which would aid in dispersal through wind or attachment to animal bodies (Sorensen, 1986; Elbaum et al., 2007; Cox, 2010). Another possible explanation is the pervasive hybridization and polyploidization events in this group (Kim et al., 2014; Zhang et al., 2019, 2021, 2022; Miao et al., 2021; Wang et al., 2023). This could also explain why phylogenetic relationships in Saccharinae were more highly resolved by plastome data than by nrDNA data, and why potential conflicting phylogenetic relationships were revealed between them. The superior resolution of phylogenies based on plastome data is in contrast to that of many other plant groups, where nrDNA often performs comparable or better than plastomes (Meng et al., 2022; Wei and Zhang, 2022; Lv et al., 2023). Future studies should include nuclear genomic data to further clarify the complex evolutionary history of Saccharinae.

## CRediT authorship contribution statement

**Kai Chen:** Writing – original draft, Resources, Methodology, Investigation, Data curation, Formal analysis, Software, Visualization. **Yan-Chun Liu:** Writing – original draft, Resources, Methodology, Investigation, Data curation, Project administration, Writing – review & editing. **Yue Huang:** Resources, Methodology, Investigation, Data curation, Formal analysis, Software, Visualization, Writing – original draft. **Xu-Kun Wu:** Resources, Methodology, Investigation, Data curation. **Hai-Ying Ma:** Resources, Project administration. **Hua Peng:** Writing – review & editing, Conceptualization, Investigation, Project administration, Resources, Supervision. **De-Zhu Li:** Writing – review & editing, Supervision, Conceptualization, Funding acquisition, Investigation, Methodology, Project administration, Resources. **Peng-Fei Ma:** Writing – review & editing, Supervision, Conceptualization, Funding acquisition, Investigation, Methodology, Project administration, Resources.

## Data availability

All the data matrices assembled and related phylogenetic and biogeographic results in this study have been deposited to the public database Figshare (https://doi.org/10.6084/m9.figshare.28094324). The raw sequencing data have been submitted to the National Genomics Data Center (NGDC) under the project number PRJCA034246.

## Declaration of competing interest

No conflict of interest exits with the submission of this manuscript, and it is approved by all authors for publication.
