# Supplementary material for: Reassessing the phylogenetic relationships of Pseudosorghum and Saccharinae (Poaceae) using plastome and nuclear ribosomal sequences
Source: Plant Divers. 2025 Mar 15;47(3):382–93. doi: 10.1016/j.pld.2025.03.002 (PMC12146866; doi:10.1016/j.pld.2025.03.002)
Supplement: Multimedia component 2 [file mmc2.docx]

# **Supporting information**

Table S1. Taxon sampling in this study.

Table S2. Detail information of the samples of *Pseudosorghum* used in this study.

Table S3. The minimum χ2 values and their corresponding smooth values obtained from ten-fold cross-validation tests.

Table S4. The history of collected specimens of *Pseudosorghum* in China.

Fig. S1. Distribution of *Pseudosorghum* in China. ● represents the collection locations of preserved specimens, while ▲ represents the locations where we collected *Pseudosorghum* recently. Note: This map is based on the standard map of GS (2019)3266, downloaded from the standard map service website of the Ministry of Natural Resources. The base map is not modified.

Fig. S2. Plastome map of *Pseudosorghum*. The size of the plastomes ranged from 140,347 bp to 140,561 bp. The inner circle displays the four main components of the plastomes, and the dashed, gray area denotes the GC content. Genes outside and inside the outer circle are transcribed clockwise and counterclockwise, respectively. Genes belonging to different functional groups are color-coded.

Fig. S3. Bayesian tree based on the whole plastome. Only BPP values <1.0 are shown. The corresponding genera/clades are labeled by different colors following Fig. 2.

Fig. S4. Phylogenetic trees based on the CDS sequences of plastome and inferred by the ML (A) and BI (B) analyses. Only MLBS values <100% or BPP values <1.0 are shown. The corresponding genera/clades are labeled by different colors following Fig. 2.

Fig. S5. Phylogenetic trees based on the LSC region of plastome and inferred by the ML (A) and BI (B) analyses. Only MLBS values <100% or BPP values <1.0 are shown. The corresponding genera/clades are labeled by different colors following Fig. 2.

Fig. S6. Phylogenetic trees based on the SSC region of plastome and inferred by the ML (A) and BI (B) analyses. Only MLBS values <100% or BPP values <1.0 are shown. The corresponding genera/clades are labeled by different colors following Fig. 2.

Fig. S7. Phylogenetic trees based on the IR region of plastome and inferred by the ML (A) and BI (B) analyses. Only MLBS values <100% or BPP values <1.0 are shown. The corresponding genera/clades are labeled by different colors following Fig. 2.

Fig. S8. Haplotype network based on plastid DNA sequences of Saccharinae and the *Eulalia* Clade III. The stepwise mutations between haplogroups are represented as comb-like short lines on the branches. Red numbers indicate a large genetic distance (>100) in the haplotype network.

Fig. S9. Splits network analysis based on plastome with hamming distances ambiguous states and neighbor net as parameters, which indicates the reticulate evolution of the main lineages. The corresponding genera/clades are labeled by different colors following Fig. 2.

Fig. S10. Bayesian phylogenetic tree based on the nrDNA sequences. Only BPP values <1.0 are shown. The corresponding genera/clades are labeled by different colors following Fig. 2.

Fig. S11. Phylogenetic trees based on the ITS sequences and inferred by the ML (A) and BI (B) analyses. Only MLBS values <100% or BPP values <1.0 are shown. The corresponding genera/clades are labeled by different colors following Fig. 2.
